# The Group Delay and Suppression Pattern of the Cochlear Microphonic Potential Recorded at the Round Window

**DOI:** 10.1371/journal.pone.0034356

**Published:** 2012-03-28

**Authors:** Wenxuan He, Edward Porsov, David Kemp, Alfred L. Nuttall, Tianying Ren

**Affiliations:** 1 Department of Otolaryngology and Head and Neck Surgery, Oregon Hearing Research Center, Oregon Health and Science University, Portland, Oregon, United States of America; 2 UCL Ear Institute, University College, London, United Kingdom; University of Salamanca- Institute for Neuroscience of Castille and Leon and Medical School, Spain

## Abstract

**Background:**

It is commonly assumed that the cochlear microphonic potential (CM) recorded from the round window (RW) is generated at the cochlear base. Based on this assumption, the low-frequency RW CM has been measured for evaluating the integrity of mechanoelectrical transduction of outer hair cells at the cochlear base and for studying sound propagation inside the cochlea. However, the group delay and the origin of the low-frequency RW CM have not been demonstrated experimentally.

**Methodology/Principal Findings:**

This study quantified the intra-cochlear group delay of the RW CM by measuring RW CM and vibrations at the stapes and basilar membrane in gerbils. At low sound levels, the RW CM showed a significant group delay and a nonlinear growth at frequencies below 2 kHz. However, at high sound levels or at frequencies above 2 kHz, the RW CM magnitude increased proportionally with sound pressure, and the CM phase in respect to the stapes showed no significant group delay. After the local application of tetrodotoxin the RW CM below 2 kHz became linear and showed a negligible group delay. In contrast to RW CM phase, the BM vibration measured at location ∼2.5 mm from the base showed high sensitivity, sharp tuning, and nonlinearity with a frequency-dependent group delay. At low or intermediate sound levels, low-frequency RW CMs were suppressed by an additional tone near the probe-tone frequency while, at high sound levels, they were partially suppressed only at high frequencies.

**Conclusions/Significance:**

We conclude that the group delay of the RW CM provides no temporal information on the wave propagation inside the cochlea, and that significant group delay of low-frequency CMs results from the auditory nerve neurophonic potential. Suppression data demonstrate that the generation site of the low-frequency RW CM shifts from apex to base as the probe-tone level increases.

## Introduction

**Figure 1:**
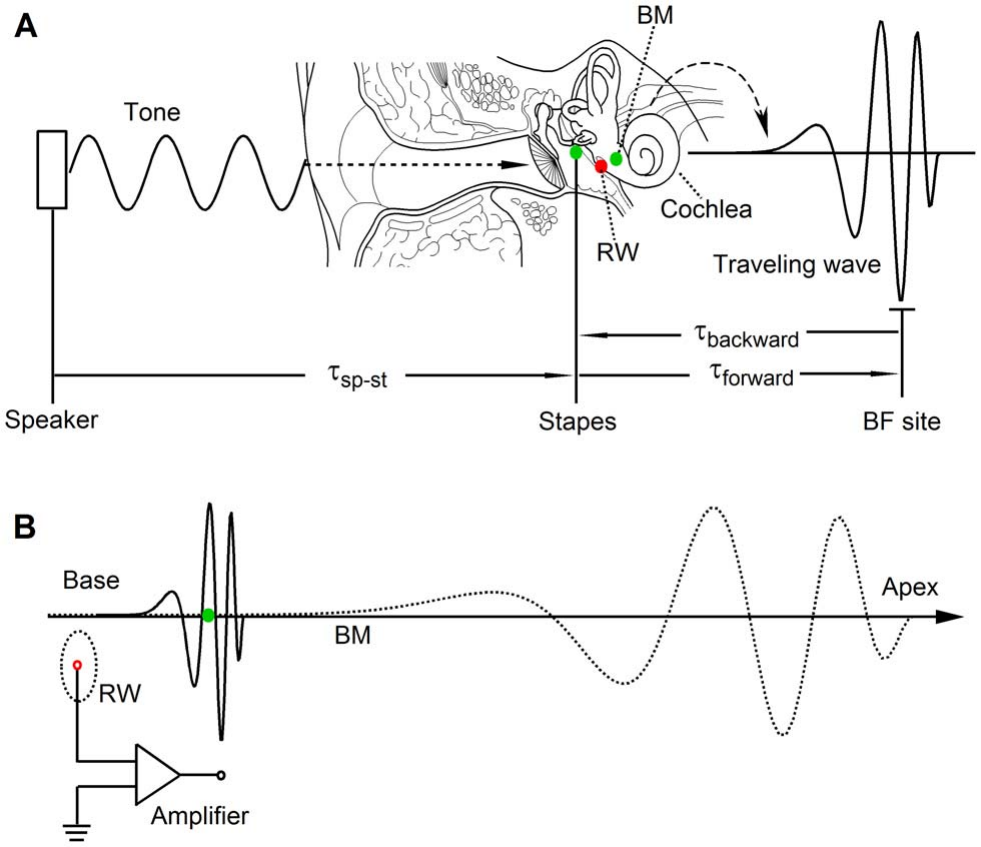
Diagram for measuring RW CM, the stapes and BM vibrations. (A) The CM was measured from the RW niche (red dot). Sound-induced vibrations were measured from the stapes and at a BM location ∼2.5 mm from the base (green dots). The temporal relationships between signals are described by the following delays: the delay from speakers to the stapes (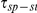), the forward and backward delays in the cochlea (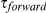 and 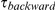). (B) The spatial relationship between travelling waves and the electrode locations for recording the RW CM. BM: the basilar membrane; RW: the round window; BF: best frequency.

When an external tone in air enters the ear canal, it vibrates the flexible eardrum and middle-ear ossicle chain resulting in a pressure change in the cochlear fluid (Fig. 1A). The pressure difference across the cochlear partition causes the basilar membrane (BM) and surrounding fluid to vibrate. Because of stiffness and mass gradients along the cochlear partition, the BM vibration starts at the cochlear base and travels toward the apex. A high frequency tone-induced vibration occurs near the base and a low-frequency vibration reaches its maximum near the apex (solid and dotted curves in Fig. 1B) [1]–[4]. The transverse BM vibration is converted into shearing motion between the tectorial membrane and the reticular lamina, which deflects the hair bundle on the top of the sensory cells toward the excitatory or the inhibitory direction dependent on the vibration direction of the BM. Hair-bundle deflection modulates conductance of mechanoelectrical transduction channels and generates receptor potential of the sensory hair cells that is recorded extracellularly as the cochlear microphonic potential (CM) [5]–[10].

The round window (RW) niche is a convenient place to record the cochlea-generated electrical responses, such as the auditory nerve compound action potential (CAP) and the CM, without intruding into the cochlea [11]–[13]. CAP has been routinely recorded to monitor cochlear sensitivity, while the application of the RW CM has been limited due to inadequate understanding of the origin of the RW CM [13]–[15]. It is commonly assumed that the RW CM is generated at the cochlear base and it has been measured for evaluating mechanoelectrical transduction process of outer hair cells (OHCs) at the cochlear base [13], [16]–[24] and for studying sound propagation inside the cochlea [25]–[27]. Further understanding of these measurements requires knowledge of the group delay and origin of the RW CM. This study quantified the intra-cochlear group delay of the RW CM by measuring the single tone-induced RW CM (red dot in Fig. 1A) and mechanical responses at the BM near the base and the stapes (green dots in Fig. 1A) before and after tetrodotoxin (TTX) application at the RW niche. The results demonstrate that the RW CM showed no significant delay at all measured frequencies above 2 kHz. The RW CM was significantly contaminated by auditory nerve neurophonic potential (ANN) at frequencies below 2 kHz. Low-level tone-induced RW CMs were effectively suppressed near the probe-tone frequency while high-level tone induced CMs were partially suppressed at high frequencies near10 kHz. Thus, we conclude that the generation location of the low-frequency RW CM varies with the stimulus level and that the phase of the RW CM does not provide temporal information on the wave propagation inside the cochlea.

## Results

**Figure 2:**
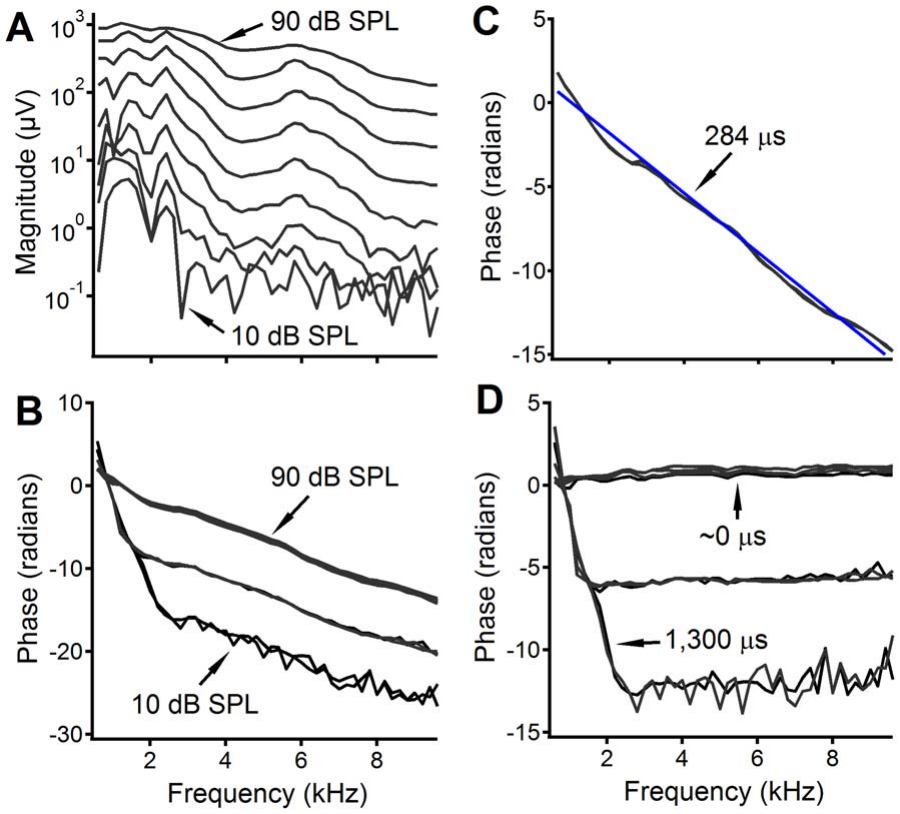
Frequency responses of the RW CM at different sound pressure levels. (A, B) Magnitude and phase of the RW CM as a function of frequency at 10 to 90 dB SPL. (C) The phase of the stapes vibration recorded at 70 and 80 dB SPL. (D) The phase of the RW CM with respect to stapes vibrations as a function of frequency. At sound levels at and above 60 dB SPL or at frequencies above 2 kHz when the sound level is ≤50 dB SPL, the phase is approximately flat, indicating a negligible group delay.

**Figure 3:**
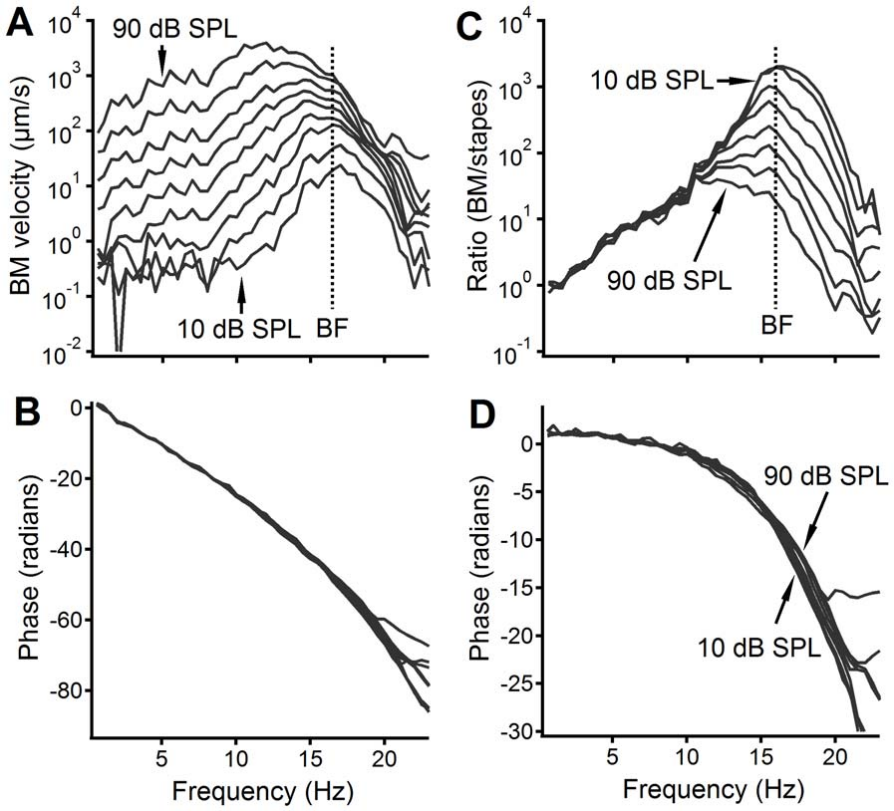
The BM vibration measured from a longitudinal location ∼2.5 mm from the cochlear base. The magnitude (A), phase (B), the magnitude ratio of the BM to the stapes (C), and phase difference between the stapes and BM (D) are presented as a function of frequency. Panels A and C show high sensitivity, sharp tuning, and nonlinear compression while panels B and D show that the phase decreases with frequency at an accelerated rate, indicating cochlear dispersion.

**Figure 4:**
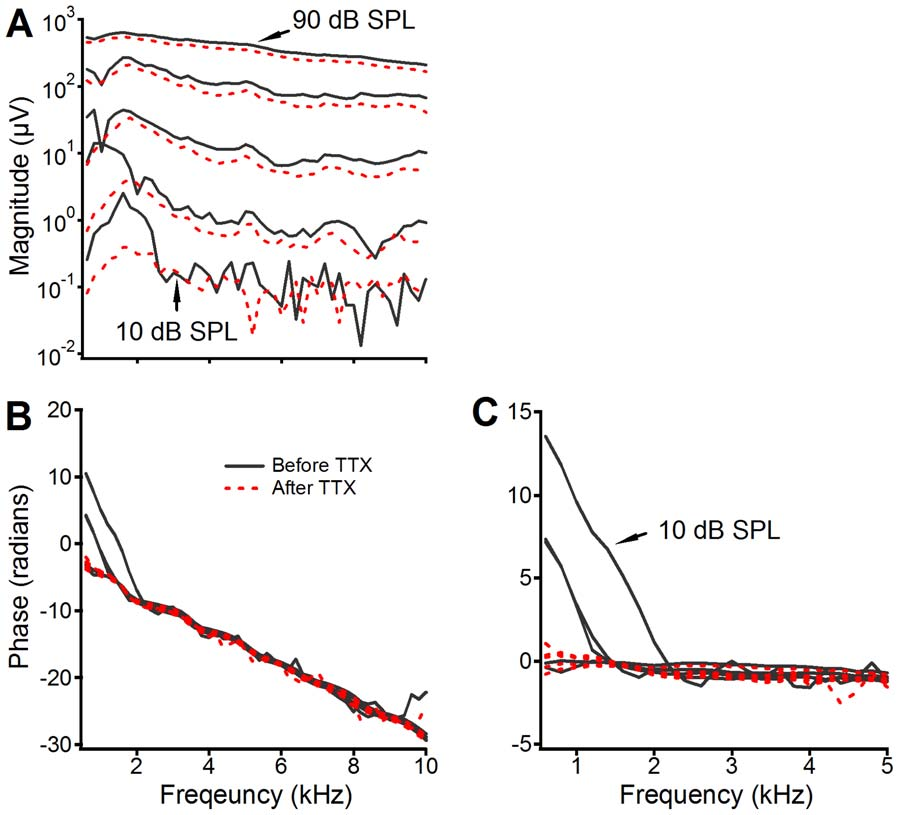
Comparison of RW CM magnitude and phase before (black solid lines) and after (red dotted lines) TTX application. Compared to solid black lines, TTX-induced CM magnitude decrease at 10 and 30 dB SPL is as much as 20 dB at frequencies below 2 kHz. Corresponding phase curves at this frequency range become less steep in panel B or flat in panel C (red dotted lines). The phase values in panel C were obtained by subtracting stapes phase from panel B.

**Figure 5:**
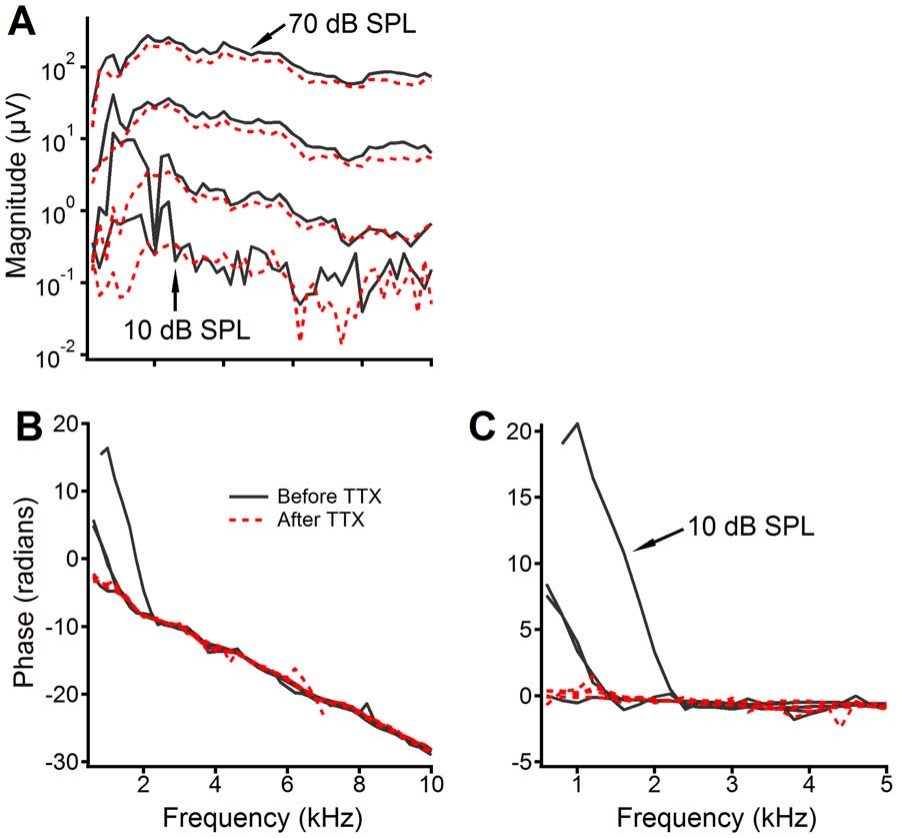
Comparison of RW CM magnitude and phase before (black solid lines) and after (red dotted lines) TTX application. Data were collected from a different sensitive cochlea. After TTX application, CMs induced by 10- and 30-dB SPL tones decreased as much as 20 dB at frequencies below 2 kHz. Panels B and C show that phase slope at low frequencies became less steep or flat (red dotted lines) after TTX application.

**Figure 6:**
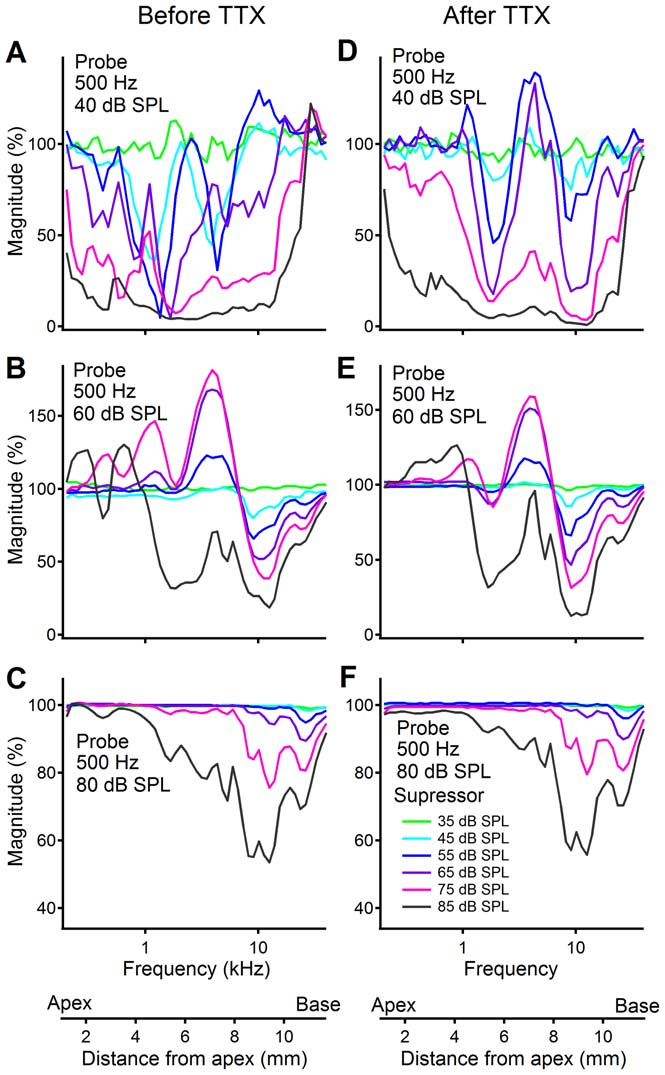
Magnitude of the RW CM as a function of suppressor frequency before (panels A, B, and C) and after (panels D, E, and F) TTX application. The RW CM was evoked by 500-Hz probe tones at 40 (panels A and D), 60 (panels B and E), and 80 (panels C and F) dB SPL, and suppressing tone levels were 35, 45, 55, 65, 75, and 85 dB SPL. For 40-dB probe tones and intermediate suppressor levels, the RW CM was suppressed mainly at ∼1.8 kHz (panels A and D) and ∼4 kHz (panel A) or ∼10 kHz (panel D). At 60-dB SPL probe level, suppression near ∼10 kHz is more significant than that near ∼1.8 kHz despite suppressor-induced CM increase at other frequencies (panels B and E). At 80-dB SPL probe-tone level, CM suppression occurred dominantly at high frequencies ≥10 kHz. After the ANN is eliminated by TTX, suppression curves in panel D become more regular than those in panel A.

**Figure 7:**
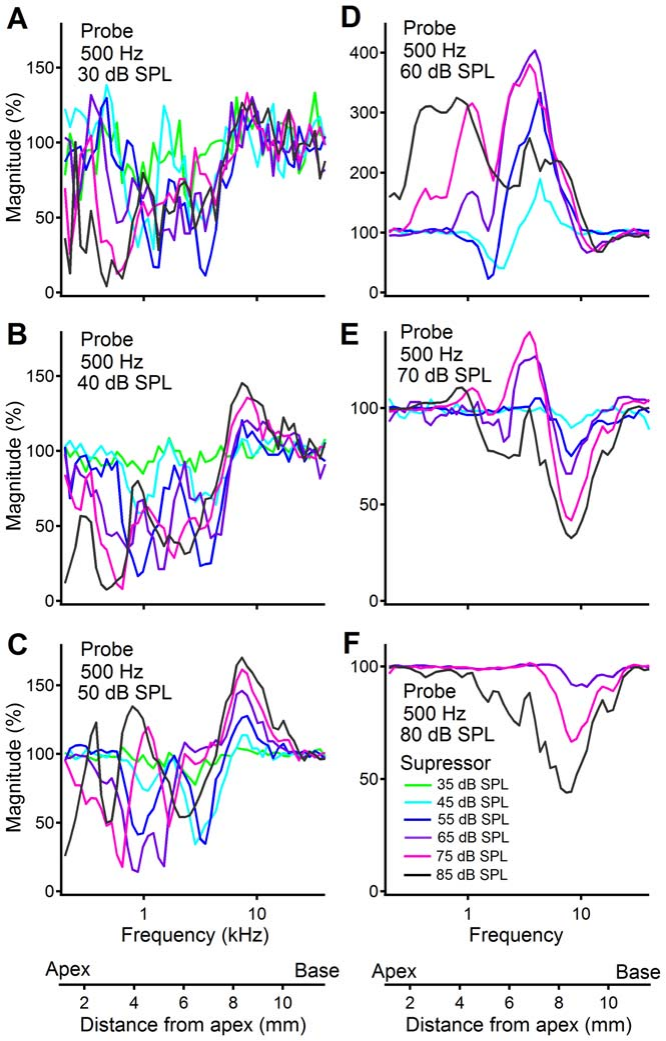
Magnitude of the RW CM as a function of suppressor frequency in a different sensitive cochlea. The RW CM was evoked by 500-Hz probe tones at 30 to 80 dB SPL, and suppressed by the second tone at different frequencies and levels. For 30-, 40-, and 50-dB probe tones and at intermediate suppressor levels, the RW CM was suppressed dominantly at frequencies below 5 kHz (panels A–C). At 70- and 80-dB SPL probe-tone levels, suppression occurred mainly at high frequencies near 10 kHz. Suppressor-induced CM increase occurred at frequencies near 9 kHz (panels B–E) and below 1 kHz (panel D).

Gerbils were used as the animal model, and all tolerated the anesthesia and surgery well. Animals showing <8-dB CAP threshold elevation near 18 kHz were considered a sensitive preparation. In addition, cochlear sensitivity was confirmed by the nonlinear compression of the BM responses. The data presented below were collected from six of 24 sensitive cochleae. Data in each figure (Fig. 2, 3, 4, 5, 6, 7) came from a single cochlea.

The magnitude and phase of the CM recorded at different sound levels and frequencies from the RW niche are shown in Figure 2. At low sound levels of 10 to 30 dB SPL (0 dB SPL is 20 µPa), CM formed a broad peak below 2 kHz and a sharp peak immediately above 2 kHz (Fig. 2A), similar to the fine structure-like appearance as shown by Henry [28]. As the sound level increased, these peaks became broader and merged together. At high sound levels above 80 dB SPL, the CM magnitude decreased slightly with frequency and showed no obvious peak. The magnitude at frequencies below 2 kHz increased nonlinearly with the sound level. At frequencies above 2 kHz, the magnitude increased proportionally with the stimulus level. The corresponding phase is presented in Figure 2B. At stimulus levels of 10 and 20 dB SPL, the phase decreased significantly with frequency, and the decrease rate became smaller above 2 kHz. For 30 to 50 dB SPL stimuli, the steep phase slope became shallow at frequencies of ∼1.2 kHz. At and above 60 dB SPL, the phase slope is approximately constant across all frequencies. Despite 6.28-radian phase difference, the phase slope above 2 kHz is approximately the same across different sound pressure levels.

As shown in Figure 1A, the CM phase lag resulted from the delay from the speaker to stapes (*τ_sp-st_*) and the delay from the stapes to the CM generation location, *i.e.*, the forward delay (*τ_forward_*). To quantify *τ_sp-st_*, the phase of the stapes vibration was measured and is presented in Figure 2C. While the overall linear phase-frequency curve indicates *τ_sp-st_* of 284 µs, the data also show an obvious phase variation different from the regression line, which likely resulted from the acoustic property of the sound system and the external and middle ear. The intra-cochlear delay was presented by the phase difference between the stapes vibration and RW CM as a function of frequency (Fig. 2D). At sound levels above 60 dB SPL, the RW CM phase is approximately flat across frequencies. The phase slopes are steep at frequencies below 2 kHz at intensities of 10 and 20 dB SPL. The group delay calculated from the flat phase slope is approximately zero and is ∼1,300 µs for the steep phase slope. Although a significant group delay was observed at low frequencies, this delay does not change with frequency, which is inconsistent with the frequency-dependent phase lag of the cochlear traveling wave [2], [29], [30].

The BM vibration measured at a longitudinal location ∼2.5 mm from the cochlea base is shown in Figure 3. Figure 3A shows the velocity magnitude of the BM vibration as a function of frequency at different stimulus levels. At low sound levels of 10 and 20 dB SPL, the response peak is at ∼16.5 kHz, *i.e.*, the best frequency (BF) of the measured BM location. The peak saturated, broadened, and shifted toward the low-frequency side (the left) as the stimulus level increased. The corresponding phase is presented in Figure 3B. Across sound pressure levels, the BM phase decreased progressively with frequency. The phase lag shown in Fig. 3B resulted from the delay from speaker to stapes 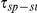 and the delay from the stapes to the BF location 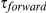. The magnitude and phase transfer function of the BM vibration were obtained and presented in Fig. 3C and D respectively. The ratio of the BM to stapes vibration velocity increased with frequency and peaked at ∼16.5 kHz at low sound levels, and decreased quickly beyond this frequency forming a sharp peak. The ratio near the peak decreased from >1,000 at 10 dB SPL to ∼10 at 90 dB SPL, indicating a ∼40 dB nonlinear compression. The response peak also broadened and shifted toward low frequencies at high sound pressure levels. Phase decreased slightly at frequencies below 10 kHz and at an accelerated rate above this frequency. At frequencies above 16.5 kHz, phase increased slightly with sound level. This typical frequency-dependent phase pattern of BM vibration is different from that of the RW CM (Fig. 2D).

To eliminate the contribution of the ANN to the RW CM, TTX was applied onto the round window membrane. After the application of TTX, the notches and the peaks of the magnitude at low frequencies below 2 kHz were reduced (Fig. 4A), indicating that the notches and the peaks likely result from the interaction between the CM and auditory nerve activities. The RW CM magnitude changes caused by the TTX application illustrated in Fig. 4 are consistent with Henry's observation [28]. At low sound pressure levels the magnitude below 2 kHz decreased up to 20 dB, indicated by the space between the solid black and dotted red lines. The steep phase slope at low frequencies became less steep or completely flat after TTX application (Fig. 4B and C). These results are consistent with data collected from a different animal (Fig. 5). Data in Figures 4 and 5 demonstrate that the observed peak response and the large group delay of the RW CM at frequencies below 2 kHz resulted predominantly from the ANN. The flat phase after the TTX application (red dotted lines in Figs. 4C and 5C) indicates that the RW CM phase does not reflect the traveling wave delay.

The suppression of a 500-Hz probe tone-induced RW CM by a second tone is presented in Fig. 6. Data were collected from a sensitive cochlea before (panels A, B, and C) and after (panels D, E, and F) the TTX application. For 40-dB SPL probe tone-evoked CM (panel A), the CM magnitude changed with the level and frequency of the suppressor (see figure legends in panel F). Suppressors at 45 and 55 dB SPL induced >50% reduction of the CM at frequencies near 1 and 4 kHz forming two notches near these frequencies. As the level of the suppressor increased, the CM magnitude decreased at a broad frequency range from 200 Hz to ∼25 kHz. At the suppressor level of 85 dB SPL, the CM was almost completely suppressed at frequencies between 2 to 3 kHz. Since a low level tone-induced BM vibration is more spatially specific than that induced by a high-level tone, the data in panel A indicate that the 40-dB SPL probe tone-induced CM was mainly generated from the cochlear location corresponding to frequencies below 5 kHz. In contrast to panel A, panel B shows that the CM decreased slightly near 2 kHz but increased significantly near 4 kHz for suppressors at and below 75 dB SPL. Suppressors at a frequency >7 kHz resulted in a significant decrease in the CM magnitude. The maximal-suppression frequency shifted toward the high frequency as the suppressor level increased (Fig. 6B). The data in panel B suggest that the 60-dB SPL probe tone-induced CM was generated mainly from the cochlear location with frequencies >7 kHz. For 80-dB SPL tone-induced CM, 45- and 55-dB suppressors suppressed the CM only at frequencies above 20 kHz (panel C). As the suppressor level increased, the maximal suppression frequency shifted toward low frequencies and resulted in the maximal decrease of ∼45% near 10 kHz.

Compared to panel A, data collected after the TTX application in panel D show two clear notches near frequencies 1.8 and 10 kHz and a peak near 4 kHz. Suppressor-induced CM decrease and increase are closely related to the suppressor level. The CM magnitude changes became less frequency specific at the suppressor levels of 75 and 85 dB SPL. Across different suppressor levels, the suppression curves of the CM in panel D are more regular than those in panel A. The irregular suppression patterns at low suppressor levels in panel A resulted likely from the contribution of the ANN. This speculation is consistent with the observation that the ANN contributes significantly to the low-frequency RW CM (Figs. 4 and 5) at low sound levels. The suppression near frequency 1.8 kHz is more significant at the probe level of 40 dB SPL (panel D) than that at the probe level of 60 dB SPL (panel E), and it almost disappears at probe level of 80 dB SPL (panel F). At 80 dB SPL of the probe level, suppression occurred dominantly at high frequencies at and above 8 kHz. The data in Fig. 6 also show that the maximal suppression decreased with the probe-tone level. A 75-dB suppressor can suppress 40-dB probe tone-induced CM by >95% (Fig. 6A and D), while it reduced 80-dB probe tone-induced CM by only ∼20% (Fig. 6C and F).

To confirm the suppression pattern observed in Fig. 6, a more detailed data set collected from a different sensitive animal is presented in Fig. 7. The RW CM was evoked by a 500-Hz probe tone at 30 to 80 dB SPL, and suppressed by an additional tone at different frequencies and sound levels. For 30-, 40-, and 50-dB probe tones and at intermediate suppressor levels, the RW CM was suppressed dominantly at frequencies below 5 kHz (panels A–C). At 70- and 80-dB SPL probe-tone levels, suppression occurred mainly at high frequencies near 10 kHz (panels E and F). Suppressor-induced CM increase occurred at frequencies near 9 kHz (panels B–E) and below 1 kHz (panel D). Despite minor differences between the two figures, the suppression pattern in Fig. 7 is consistent with that in Fig. 6.

## Discussion

In comparison to previous experiments [14], [31], [32], a major advantage in this study is that the CM and stapes vibration were measured from the same cochlea. This allows us to precisely quantify the intra-cochlear or forward delay (*τ_forward_*) from the stapes to the CM generation site by removing the propagation delay of the stimulus from the speaker to the stapes (*τ_sp-st_*), as illustrated in Fig. 1.

The main finding of this study is that, after the TTX application, the RW CM showed no significant group delay; and at low and intermediate sound levels, the low-frequency RW CM can be suppressed near the probe-tone frequency while, at high sound levels, it was suppressed only at high frequencies above 8 kHz. One may think that the lack of a significant CM group delay is consistent with the common belief that the low-frequency RW CM is generated from the cochlear base. However, since CM results from the OHCs excited by a traveling wave, the CM should show a forward delay for sound to travel from the stapes to the CM generation site even if it is generated near the cochlear base. As indicated by phase transfer function of the BM in Fig. 3, although the phase lag at the low frequency is small it progressively increases with frequency. In fact, the travel time of the cochlear-partition vibration was demonstrated electrically by Dallos and Cheatham [33] using an electrode pair in scala tympani and vestibule and by Fridberger et al. using a micro electrode recording from inside the organ of Corti [34]. These previous studies indicate that CM recording with fine spatial resolutions can reveal the travelling-wave delay. When CM is recorded remotely from the RW, the extracellular potential generated by hair cells at one location can be cancelled by those generated at other locations. Although the phase cancellation likely contributes to the phase of the RW CM, the phase cancellation may not be the only mechanism responsible for the observed flat phase.

Using a scanning laser interferometer, Ren found that the BM vibration in response to a 16-kHz tone at physiological sound levels occurs over a <1-mm region along the cochlear length [2]. The vibration phase changes rapidly near the BF location and results in a wavelength as small as ∼0.2 mm. A recording method with fine spatial resolution, such as the intracellular or differential recording, is required to detect the phase or temporal information of the CM. For the RW CM measurement, the recording electrode is often placed in the RW niche and the reference electrode is in muscles close to the bulla. Due to the large distance between the electrodes, the spatial resolution of the RW CM recording is relatively low. Compared to the inter-electrode distance, the longitudinal extent of the BM vibration is small (solid line in Fig. 1B). As the stimulus frequency decreases, the excitation area moves toward a more apical location according to the cochlear frequency-location map [35], [36]. Although the longitudinal extent of the BM vibration at a low frequency is larger than that for a high-frequency tone, the excited OHCs for a low frequency tone still located at the apical side of the electrodes (dotted curve in Fig. 1B). Moreover, the instantaneous vibration pattern of the BM in response to a low-frequency tone (dotted curve) is similar to that of a high-frequency tone (solid curve). As a result, the phase of the summed extracellular potential can remain constant, as shown by Figs. 2, 4 and 5.

It has been shown that the longitudinal pattern of the BM vibration in response to tone changes with the stimulus level [2], [37], [38]. As the sound pressure increases, the vibration pattern extends toward the cochlear base. Due to the nonlinear compression near the BF location, the vibration near the BF location increases less than that near the base at high sound levels. This level-dependent change in the spatial pattern was also indirectly shown by the transfer function of the BM vibration measured from a single location (Fig. 3A), *i.e.*, the BM vibration magnitude at frequencies below 8 kHz increased by ∼40 dB more than that near the BF. Consequently, the relative contribution of the OHCs from the BF location and the cochlear base to the RW CM varies with the sound pressure level. This is consistent with suppression data which show low-frequency suppression at low probe levels (Figs. 6A, B and 7A–C) and high-frequency suppression at high probe levels (Figs. 6C, F and 7E, F). According to the frequency-location relation shown by horizontal axes of Figs. 6 and 7, suppression data indicate that low-level tone-induced RW CMs were mainly from cochlear locations near the apex, and high-level tone-induced CMs partially came from locations near the base.

According to the cochlear frequency-location map [36], the maximal suppression should occur near probe frequency (500 Hz). This apparently is not consistent with the data which show a notch near 1.8 kHz in Fig. 6A, D and magnitude decrease at a broad frequency range below 5 kHz (Fig. 7A–C). We speculate that the spatial relationship between the CM generators and the electrode locations probably contributed to observed complex suppression patterns. The OHCs that contributed to the RW CM the most, not those near the BF location, determined the maximal suppression. Due to the small distance between the CM generators and the recording electrode, the RW recording preferably detects the CM generated at the cochlear base. When the CM generators at the base are suppressed by high-frequency suppressor, the high-frequency notch occurs. Suppressor-induced CM increase in Fig. 6B and E and Fig. 7B–E likely results from suppression of CM components with phase different from that of recorded components, and/or from the suppressor-induced enhancement of the BM response to the probe tone.

The current data do not conflict with those reported by Patuzzi et al. [14], [31], [32]. In those experiments, a 200-Hz low-frequency tone at sound pressure level of 105 dB SPL was used to evoke the CM. A tone at such a high level saturates the mechanoelectrical transduction of the OHCs through almost the whole cochlea. This stimulus may not traumatize the cochlea as the authors pointed out, it, however, can suppress the BM response to a high frequency tone [39]. This likely complicates the interpretation of the high-level sound-induced low-frequency CM as a measure of the integrity of the mechanoelectrical transduction of the OHCs near the cochlear base. This complication can be avoided by using a relatively low-level probe tone for the CM recording. In the current study, the frequency response of the RW CM was measured at sound levels from 10 to 90 dB SPL and suppression experiment was conducted at probe-tone levels 30 to 80 dB SPL. The data in Fig. 6C and F and Fig. 7F show that the RW CM induced by an 80-dB SPL low-frequency tone is partially generated from the cochlear base, indicating that it is not necessary to use probe-tone levels above 80 dB SPL. In addition, Patuzzi et al. did not report the phase and frequency response of the RW CM [14], [31], [32]. Thus, the current study expanded the previous observation by using more physiologically relevant low probe-tone levels and by measuring frequency response of the RW CM. More importantly, the intra-cochlear group delay of the RW CM was measured in the current study.

In summary, the single tone-evoked electrical and mechanical responses were measured at the RW, the stapes, and the BM. The results demonstrate that after eliminating the ANN potential, the intra-cochlear group delay of the single tone-evoked microphonic potential recorded at the RW is approximately zero. In contrast to the BM vibration, the RW CM does not provide temporal information of traveling waves inside the cochlea. The suppression data show that, at low levels, the low-frequency RW CM comes mainly from the BF location while, at high sound levels, it partially comes from the cochlea base. These experimental findings demonstrate that the group delay of the microphonic potential recorded at the RW cannot reflect the propagation delay of the waves inside the cochlea under physiological conditions. The current data also indicate that the low-frequency RW CM may not reliably reflect the integrity of mechanoelectrical transduction channels of auditory sensory cells at the cochlear base.

## Materials and Methods

Twenty-eight young Mongolian gerbils of 50–80 g were used in this study. The animal was initially anesthetized using a combination of ketamine chloride (80 mg/kg) and xylazine (10 mg/kg) as described previously [2], [29]. Supplemental anesthetic of half the initial dose was given about every hour depending on anesthesia depth. All animals were tracheotomized; a ventilation tube was used to maintain natural breathing. A heating blanket was used to keep body temperature at ∼38°C. Animal use protocol for this study (IS00000130) was approved by the Oregon Health & Science University Institutional Animal Care and Use Committee.

The left external ear was partially removed and a plastic speculum coupled to two electrostatic speakers (EC1, Tucker-Davis Technologies, Alachua, FL) and a sensitive microphone (10B+, Etymotic Research, Inc., Elk Grove Village, IL) coupled to the bony external ear canal to form a closed sound field. The bulla was opened through a ventrolateral approach; the RW and stapes were exposed. A silver electrode was placed in the RW niche to record CM and CAP. The reference electrode was placed in muscle close to the bulla.

A LabVIEW-based (National Instruments, Austin, TX) program was used to control TDT hardware (System 2, Tucker-Davis Technologies, Alachua, FL) for signal generation and data acquisition. The analogue electrical signals from D/A converters were used to drive the speakers, and the sound pressure levels were controlled using two programmable attenuators. For the single-tone stimulation, the acoustic tone bursts with 20-ms duration and 1-ms rise and fall time were generated at frequencies from 0.2 to 10 kHz with 0.2-kHz steps and at sound levels from 10 to 90 dB SPL with 10 dB per step.

BM and stapes vibrations were measured using a heterodyne laser interferometer as described previously [40]–[42]. The phase of vibrations was referenced to the electrical signal that drove the speaker. The electrical signal from the RW was amplified 2,000 times with a frequency bandwidth from 100 Hz to 50 kHz using a bioamplifer (CWE Super-Z, BMA-200). The signals from the interferometer controller and bioamplifier were averaged 20–100 times according to the signal-to-noise ratio. The magnitude and phase of the averaged signals at different frequencies and intensities were derived through the Fourier transform. The group delay was calculated from the slope of the linear regression line of the phase-frequency data according to the equation: *τ = −Δφ/2πΔf,* where *τ* is the group delay in seconds, *Δφ* is the phase difference in radians; *Δf* is frequency difference in Hz. The phase difference between the stapes vibration and RW CM was obtained by subtracting the stapes phase from the RW CM phase.

To eliminate the ANN, ∼40 µl of 20-µM TTX (Sigma, St. Louis, MO) was applied into the RW niche. After TTX application, the CAP was monitored continuously, and the disappearance of the CAP indicated the effectiveness of TTX [43].

In order to determine the origin of the RW CM, suppression of the low-frequency RW CM was observed by presenting a probe tone and a pure-tone suppressor simultaneously. The 500-Hz probe tone was presented at sound pressure levels from 30 to 80 dB SPL. When the magnitude and phase of the CM were continuously recorded, suppressors at frequencies from 0.2 to 40 kHz at sound pressure levels 35, 45, 55, 65, 75, or 85 dB SPL were sequentially presented. Suppression was presented by the magnitude of the CM as a function of frequency of the suppressor. The CM magnitude was normalized to the level without suppressor. The contribution of the neural activity of the auditory nerve to the suppression was evaluated by collecting data before and after TTX application.
